# Improving clinical management of colon cancer through CONNECTION, a nation-wide colon cancer registry and stratification effort (CONNECTION II trial): rationale and protocol of a single arm intervention study

**DOI:** 10.1186/s12885-020-07236-y

**Published:** 2020-08-18

**Authors:** I. van den Berg, S. van de Weerd, J. M. L. Roodhart, G. R. Vink, R. R. J. Coebergh van den Braak, C. R. Jimenez, S. G. Elias, D. van Vliet, M. Koelink, E. Hong, W. M. U. van Grevenstein, M. G. H. van Oijen, R. G. H. Beets-Tan, J. H. J. M. van Krieken, J. N. M. IJzermans, J. P. Medema, M. Koopman, on behalf of the CONNECTION-study group

**Affiliations:** 1 Department of Surgery, Erasmus MC, University Medical Center Rotterdam, Rotterdam, the Netherlands; 2 Department of Medical Oncology, University Medical Center Utrecht, Utrecht University, Utrecht, the Netherlands; 3 Laboratory for Experimental Oncology and Radiobiology, Center for Experimental and Molecular Medicine, Cancer Center Amsterdam, Amsterdam UMC, University of Amsterdam, Meibergdreef 9, 1105 AZ Amsterdam, The Netherlands; 4 Department of Pathology, Radboud University Medical Centre, Nijmegen, the Netherlands; 5 Oncode Institute, Amsterdam UMC, University of Amsterdam, Amsterdam, The Netherlands; 6 Netherlands Comprehensive Cancer Organisation, department of research, Utrecht, the Netherlands; 7 Department of Medical Oncology, Amsterdam UMC- location VUmc, Amsterdam, the Netherlands; 8 Julius Center for Health Sciences and Primary Care, University Medical Center Utrecht, Utrecht University, Utrecht, The Netherlands; 9 Department of radiology, The Netherlands Cancer Institute, Amsterdam, The Netherlands; 10 Department of Surgery, University Medical Center Utrecht, Utrecht University, Utrecht, the Netherlands

**Keywords:** Colon cancer, Consensus molecular subtypes, Neoadjuvant chemotherapy, Surgery

## Abstract

**Background:**

It is estimated that around 15–30% of patients with early stage colon cancer benefit from adjuvant chemotherapy. We are currently not capable of upfront selection of patients who benefit from chemotherapy, which indicates the need for additional predictive markers for response to chemotherapy.

It has been shown that the consensus molecular subtypes (CMSs), defined by RNA-profiling, have prognostic and/or predictive value. Due to postoperative timing of chemotherapy in current guidelines, tumor response to chemotherapy per CMS is not known, which makes the differentiation between the prognostic and predictive value impossible. Therefore, we propose to assess the tumor response per CMS in the neoadjuvant chemotherapy setting. This will provide us with clear data on the predictive value for chemotherapy response of the CMSs.

**Methods:**

In this prospective, single arm, multicenter intervention study, 262 patients with resectable microsatellite stable cT3–4NxM0 colon cancer will be treated with two courses of neoadjuvant and two courses of adjuvant capecitabine and oxaliplatin. The primary endpoint is the pathological tumor response to neoadjuvant chemotherapy per CMS. Secondary endpoints are radiological tumor response, the prognostic value of these responses for recurrence free survival and overall survival and the differences in CMS classification of the same tumor before and after neoadjuvant chemotherapy. The study is scheduled to be performed in 8–10 Dutch hospitals. The first patient was included in February 2020.

**Discussion:**

Patient selection for adjuvant chemotherapy in early stage colon cancer is far from optimal. The CMS classification is a promising new biomarker, but a solid chemotherapy response assessment per subtype is lacking. In this study we will investigate whether CMS classification can be of added value in clinical decision making by analyzing the predictive value for chemotherapy response. This study can provide the results necessary to proceed to future studies in which (neo) adjuvant chemotherapy may be withhold in patients with a specific CMS subtype, who show no benefit from chemotherapy and for whom possible new treatments can be investigated.

**Trial registration:**

This study has been registered in the Netherlands Trial Register (NL8177) at 11–26-2019, https://www.trialregister.nl/trial/8177. The study has been approved by the medical ethics committee Utrecht (MEC18/712).

## Background

Colon cancer is one of the most common types of cancer in the Netherlands with an incidence of around 9.800 patients in 2018 [1]. Approximately 80% of patients present with local disease (stage I-III). Curative surgery followed by adjuvant systemic chemotherapy is standard of care in patients with microsatellite stable (MSS) high-risk stage II and stage III colon cancer. Despite this intensive treatment, 20–30% of the patients develop metastatic disease. These patients do not benefit enough from the current adjuvant systemic therapy. Moreover, it is estimated that 50% will not develop metastases after surgery alone and are therefore over-treated with adjuvant chemotherapy. Identifying the patients at risk of developing metastases, as well as those responding to therapy is a clear unmet need in colon cancer care. The development of new prognostic and predictive markers for chemotherapy response is therefore of utmost importance.

Many efforts have been undertaken to stratify CRC patients into biologically and clinically distinct subtypes. One of these led to the development of the Consensus Molecular Subtypes (CMSs), which is based on RNA expression profiling of tumor tissue and which is currently considered to be the most robust molecular stratification in CRC [2]. CMS1 is characterized by hypermutation, microsatellite instability (MSI) and strong immune infiltration. CMS2, the canonical subtype, has marked WNT and MYC signaling activation. CMS3 is enriched for *KRAS*-mutations and shows evident metabolic deregulation. CMS4, the mesenchymal subtype, is characterized by prominent TGF- activation, stromal invasion and angiogenesis activation. Subtyping in a large heterogeneous patient cohort (*n* = 2.129) with stage I to IV colorectal cancer showed significant differences in prognosis, with CMS4 as the poor-prognosis subtype, confirming the clinical relevance of the intrinsic processes implicated in each CMS [2].

These results support the idea that the CMSs might have predictive value for response to chemotherapy. Due to the postoperative timing of chemotherapy in current guidelines, the tumor response to chemotherapy is not assessable, which makes a distinction between the prognostic value and predictive value of the subtypes impossible. Only a randomized controlled trial in which patients would be randomized in either surgery plus adjuvant chemotherapy or surgery alone would make this distinction possible. However, this causes ethical dilemmas because chemotherapy would be withhold in patients who might actually benefit. Yet, a solid response assessment per subtype is necessary for implementation in clinical decision-making. We therefore propose to treat patients with two neoadjuvant and two adjuvant courses of CAPOX and determine the response in tumor resected specimens.

Applying neoadjuvant chemotherapy may have several advantages: the possibility of response monitoring, early eradication of micrometastases and more complete resections. Neo-adjuvant treatment is already standard of care for different GI malignancies including esophageal, gastric and rectal cancers [3–7]. The FOxTROT Collaborative Group (2012) was the first to set up a neoadjuvant trial in patients with locally advanced resectable colon cancer and concluded that preoperative chemotherapy is feasible with acceptable toxicity and perioperative morbidity [8]. After this pilot study, they conducted a randomized phase 3 trial investigating the effect of neoadjuvant chemotherapy in patients with a cT3–4 N0-2 M0 colon cancer. Patients were randomized 2:1 between 6 weeks of neoadjuvant combined with 18 weeks of adjuvant FOLFOX/CAPOX or 24 weeks of adjuvant FOLFOX/CAPOX. Neoadjuvant chemotherapy was safe with less major surgical complications, significant down-staging and a reduced risk of incomplete resection. Although the primary endpoint of the study (freedom from recurrent or persistent disease after 2 years) was not met, the risk of a recurrence after 2 years was reduced to 13.6% with peri-operative chemotherapy compared to 17.2% with adjuvant chemotherapy only (HR 0.75 (0.55–1.04), *p* = 0.08)) [9].

In the proposed study we will investigate the predictive value of the CMS classification on chemotherapy response in a neoadjuvant setting, including pathological response and radiological response and their correlation with RFS and OS. This allows us to determine therapy efficacy in individual patients and per subtype.

### Objective

The primary aim of this study is the evaluation of the pathological tumor response to neoadjuvant systemic chemotherapy per CMS in patients with MSS high risk stage II and stage III colon cancer.

## Methods

### Study design

CONNECTION II is a prospective, multicenter interventional cohort study that will be performed as a substudy of the Prospective Dutch ColoRectal Cancer cohort (PLCRC). PLCRC is a nationwide cohort study of the Dutch Colorectal Cancer Group (DCCG), facilitating scientific research to improve the outcome and quality of life of patients with colorectal cancer [10]. We aim to include patients in 8–10 Dutch hospitals that participate in PLCRC.

**Fig. 1:**
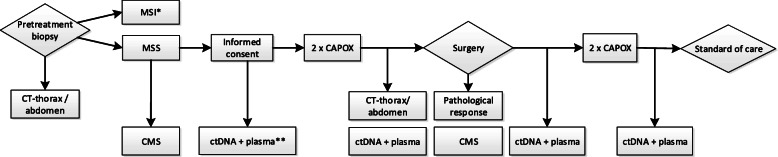
Flow diagram of clinical study. *Patients with an MSI tumor will be excluded from this study. ** At 4 time points blood samples will be collected for ctDNA analyses and future biomarker studies

In CONNECTION II patients with a MSS cT3–4NxM0 colon tumor will be treated with two courses of neoadjuvant and two courses of adjuvant capecitabine and oxaliplatin (CAPOX) (Fig. 1 and Table 1). The CMS classification will be determined on both the pre-treatment biopsies and the resection specimen. At least 4 multi-region biopsies will be taken pre-treatment to ensure a sample with vital tumor and sufficient RNA quality. Tumor response will be assessed on the resection specimen using the tumor response grading (TRG) system as proposed by Dworak et al. [11]. Radiological response evaluation will be centrally performed by dedicated radiologists on sequential CT scans made at baseline and after two courses of neoadjuvant chemotherapy but before resection. Pathologists and radiologists will be blinded for the CMS classification.

**Table 1:** Study flowchart of clinical study

Study procedures	Inclusion	Neo-adjuvant Chemotherapy							Surgery				Adjuvant Chemotherapy						Follow up			
*week*	< 0	1	2	3	4	5	6	7	8	9	10	11	12	13	14	15	16	17	18	19	20	21
Check in- and exclusion	x																					
Sign Informed Consent	x																					
Blood withdrawal for ctDNA + plasma	*x* ^*a*^							*x* ^*b*^				*x* ^*c*^									*x* ^*d*^	
CT-scan	x							*x* ^f^														
Surgery									x^g^													
CAPOX		*C1D1*			*C2D1*								*C3D1* ^*e*^			*C4D 1*						
Record medical history	x	x			x								x			x						
Document concomitant medication/therapies	x	x			x								x			x						

^a^: blood withdrawal may be done at screening or immediately before cycle 1 day 1

^b^: blood withdrawal to be performed after cycle 2 week 3 and before surgery

^c^: blood withdrawal to be performed before cycle 3 day 1

^d^: blood withdrawal to be performed approximately 12 weeks after surgery

^e^: Cycle 3 day 1 should ideally start within 4–8 weeks after surgery, at the latest: 12 weeks after surgery

^f^: CT should be performed after completion of cycle 2 and before surgery

^g^: Surgery should ideally be performed 7–9 weeks after Cycle 1 day 1, but has to be performed 11 weeks after Cycle 1 day 1

Optionally, blood samples are taken for circulating tumor DNA (ctDNA) analysis and plasma storage at four time points: at baseline, after neoadjuvant treatment, after surgery and after completion of the adjuvant chemotherapy. Follow-up will be performed until 10 years post-surgery. Data on local recurrences, metastases and survival will be documented.

### Study population

Patients diagnosed with resectable cT3–4NxM0 colon cancer are eligible for the CONNECTION II trial. Baseline CT-scans of all patients will be reviewed by dedicated radiologists in the treating hospitals with special focus on tumor staging. MSI status will be determined on biopsy material to exclude patients with an MSI tumor [12].

Patients are eligible when they meet the following criteria:

- Able and willing to provide written informed consent for the CONNECTION II study
- Informed consent signed for PLCRC components ‘clinical data’ and ‘future studies’
- MSS based on pre-treatment biopsy by immunohistochemistry (IHC)
- Fit to undergo neoadjuvant chemotherapy with capecitabine + oxaliplatin and subsequent surgery judged by the primary treating physician
- Adequate bone marrow, liver and renal function

Patients will be excluded if any of the following criteria are met:

- Any other malignant disease within the preceding 5 years apart from non-melanotic skin cancer, carcinoma in situ and early stage disease with a recurrence risk of less than 5%
- Colonic obstruction that cannot be defunctioned by a stoma
- Pregnant or lactating women

### Main study parameter/endpoint

The primary endpoint is the pathological tumor response to neoadjuvant chemotherapy per CMS. The pathological response will be centrally scored on HE-stained slides from the resection specimen using the tumor response grading system according to Dworak [13, 14]. Based on results from the FOxTROT study, a good response will be defined as TRG2, TRG3 or TRG4; poor response as TRG1 or TRG0. The CMS classification will be determined on the pre-treatment biopsies and on the resection specimens. RNA will be isolated from FFPE material and analyzed on the nCounter SPRINT profiler, a reliable and robust platform for samples with degraded RNA such as FFPE samples [12, 15, 16].

### Secondary study parameters/endpoints

- Additional pathological markers to assess the tumor response: the modified Ryan scheme (TRS) [13] and expression of Ki-67 and Caspase-3 and morphological cytostatic-cytotoxic effects on HE-stained tissue slides.
- Pathological response per TRG and TRS category separately for the different CMS subtypes.
- Radiological tumor response to neoadjuvant chemotherapy.
- Recurrence free survival (RFS) at two and three years. RFS is defined as the time elapsed between the diagnosis of the primary tumour and either the date of any recurrence of disease, time of death, or the date of the last follow-up visit at which a patient was considered to have no recurrence.
- Overall survival (OS) at five and ten years.
- Therapy-induced CMS differences.
- Prognostic and predictive value of cytotoxic lymphocytes (CytoLym) and cancer-associated fibroblasts (CAF) infiltration scores.
- Diagnostic accuracy of ctDNA measurements for monitoring treatment response to neoadjuvant treatment and detection of residual disease.
- Exploration of proteome profiles for monitoring treatment response to neoadjuvant treatment and detection of residual disease.
- Percentages of pathological complete (R0), pathological microscopic incomplete (R1) and pathologically macroscopic incomplete (R2) resections.
- Surgical complication rate (i.e. wound infections and anastomotic leak).

### Statistical analysis

#### Primary study endpoint

The primary study endpoint is the pathological tumor response per CMS using the TRG system according to Dworak [11]. Pathologic tumor regression rates with corresponding 95% confidence intervals will be analyzed per CMS subgroup using the Wilson Method.

#### Secondary study endpoints

Categorical data (pathological tumor response according to the Modified Ryan scheme) are compared using Chi-square analysis or Fisher’s exact test and are shown as numbers, relative and absolute rates. Continuous data (CytoLym and CAF infiltration scores, radiological tumor response, pathological response by percentage of Ki-67 and Caspase-3 positive neoplastic cells) are compared using non-parametric T-test or Mann-Whitney U test where appropriate and are shown as mean and standard deviation or median and interquartile range (25–75%). *P*-values are two-tailed and results < 0.05 are considered significant.

The OS at 5 and 10 years and RFS at 2 and 3 years will be calculated and depicted by means of the Kaplan Meier technique and will be compared using the (stratified) logrank test. Hazard ratios and 95% confidence intervals will be calculated with a (stratified) cox-proportional hazard analysis. The RFS will be analyzed per CMS subgroup, per TRG and radiological response. All estimates will be accompanied by 95% confidence intervals.

#### Sample size calculation

We based our sample size calculation on the desired precision with which we will be able to estimate the pathological response rates to neoadjuvant chemotherapy within each CMS subtype. This precision is quantified by the margin of error (the radius of the 95% confidence interval), which we set at a maximum of 15%. This margin of error is achieved with 35 patients in the least prevalent CMS subgroup, namely CMS3, and an anticipated 11 pathologic responses, yielding a response rate of 31% with a 95%CI of 19–48%. Based on the currently observed ratios of subtypes derived from the large consensus dataset after exclusion of the MSI tumors (which holds CMS1 tumors for most part) we will need a total of 209 MSS patients (CMS2 49%, CMS3 17%, CMS4 35%). With this sample size we anticipate maximum margins of error of 8.9, 14.7, and 10.3% for CMS2, CMS3, and CMS4 respectively, and 6.2% overall.

The above depends on the assumption that the response rates will not be higher than ~ 30% within each CMS subgroup. If response rates will actually be closer to 50%, the maximum margin of error will increase.

The sample size hence indicates that for the analysis, 209 patients will be needed for whom follow-up and subtype is known. We expect a 25% loss in patients due to loss of follow-up, insufficient quality of the biopsy material or failure to faithfully assign patients to a subgroup based on the RNA expression profiles resulting in a total of 262 patients needed to have sufficient data for both the primary and secondary outcomes.

### Data collection and data management

Data collection and data management will be performed by the Netherlands Comprehensive Cancer Organization (IKNL). They have broad experience with continuous data collection based on high quality electronic case report forms (eCRFs) which guarantees complete and timely recording, handling and storage of data and documents. All local and central data managers are registered and the electronic database (TRIAS) is ISO certified. Data will be documented in line with ‘Good Clinical Practice (GCP)’ and Dutch legal requirements. Major violations of the protocol will be recorded.

### Monitoring

No data and safety monitoring board (DSMB) will be assigned, since patients are subjected to an intervention with a low postoperative morbidity that is already being performed in routine clinical practice. No interim analyses will be performed.

### Auditing

Independent monitoring of the study is performed by a qualified monitor of IKNL. The monitor plan is based on the judgement of the IRB that study participation is of low to moderate risk. Monitoring will be performed by investigating the electronic trial database and performing site visits. Each participating site will be visited at least once, with repeat visits to sites where performance is a concern. The quality assessment will focus on the safety, wellbeing and rights of the patients, the quality of the documented data in the eCRF and their traceability to source documents and the completeness of the regulatory binder. After each monitor visit, the trial monitor reports feedback to the project leader, study coordinator and local investigator.

### Adverse events

The treatment with CAPOX in this study is standard of care, therefore AE and SAE are not expected to be different. As both the treatment with CAPOX and the surgery are part of the standard of care, only two specific SAEs are defined which are possibly related to the adjusted study schedule. Information will be collected on patients who prematurely stop chemotherapy treatment and of patients who are not able to undergo planned surgery due to progressive disease/obstruction.

The following two SAEs will be reported:

- If the surgery has to be postponed for more than 8 weeks after the start of cycle 2 of CAPOX.
- If patients can not complete all the neoadjuvant chemotherapy courses.

The study coordinator will report these SAEs to the accredited Institutional Review Board (IRB) that approved the study protocol.

## Discussion

Colon cancer is one of the most common types of cancer in the Netherlands. The standard of care for patients with MSS high risk stage II and stage III colon cancer currently consists of surgery followed by systemic chemotherapy. Patient selection for adjuvant chemotherapy is still far from optimal. Approximately 50% would never develop metastases after surgery alone and is therefore over-treated with adjuvant chemotherapy. Moreover, 20–30% still develop metastatic disease despite this intensive treatment, leaving merely 15–30% that in fact benefit from adjuvant chemotherapy. This illustrates the evident need for additional predictive markers for chemotherapy benefit.

One potential marker is the CMS classification, which is based on the integration of six different molecular classification systems based on RNA expression profiling. The CMS classification divides CRC patients into four subtypes with distinctive biological features. Guinney et al. showed a clear relapse free survival and overall survival advantage for CMS1–3 compared to CMS4 in a heterogeneous patient cohort with stage I-IV CRC with divergent treatment schemes [2].

Besides the prognostic value, literature provides some support for a predictive value of CMS for response to systemic treatment. In a retrospective analysis of the NSABP C-07 trial on patients (*n* = 1033) with stage III colon cancer, only CMS2 was associated with benefit from oxaliplatin treatment, patients with CMS4 tumors did not benefit from addition of oxaliplatin treatment [14]. The mesenchymal subtype showed no benefit from 5-FU monotherapy compared to no systemic therapy in a non-randomized retrospective analysis of 222 stage III CRC patients [17].

Although being a promising molecular marker, a solid chemotherapy response assessment per subtype has not been performed and it remains unknown whether the difference in long-term outcome between CMS1–3 and CMS4 originates from differences in prognosis or response to therapy.

This makes it impossible to know whether patients with the poor-prognosis subtype (CMS4) have an impaired survival due to the aggressive nature of the tumor or due to a limited response to chemotherapy. Therefore, it is unknown whether these patients should receive chemotherapy or not. This also holds true for the other subtypes. Although CMS1–3 show superior outcomes to CMS4, it is unknown whether this is due to a favorable tumor biology or due to a substantial response to chemotherapy. We therefore believe that a solid chemotherapy response assessment per subtype is an important and essential step to distinguish between prognosis and prediction, and to incorporate the CMSs in clinical decision-making.

Administering neoadjuvant chemotherapy in the suggested study population was proven safe and feasible in the FOXTROT study [8, 9]. Importantly, the pathological tumor response was evidently associated with recurrence free survival. Patients with a complete response (TRG4) developed no recurrences after 5 years of follow-up, compared to 26% of patients that showed no regression at all (TRG0) [9]. This illustrates that the response to chemotherapy of the primary tumor may indeed be a reliable measurement for chemotherapy efficiency.

The primary endpoint of the proposed study is the pathological tumor response, which will be centrally scored using the TRG by Dworak, a highly reproducible scoring system which is often used and clinically meaningful [11]. Evidently, tumor response monitoring using histology requires invasive procedures. As a secondary endpoint, radiological response will be scored by a central board of radiologists and compared to the pathological tumor response to evaluate this noninvasive technique as a response modality. Both the histological and radiological response will be correlated to RFS and OS to assess their prognostic value.

The proposed neoadjuvant approach requires reliable clinical TNM staging to minimize the risk of overtreating patients with stage I or low risk stage II colon cancer. A meta-analysis analyzing the accuracy of T and N staging on CT imaging showed that T1–2 can be reliably distinguished from T3–4 (sensitivity 96% and specificity 70%), while nodal involvement is unreliable with a pooled sensitivity and specificity of 78 and 68% respectively [18]. Therefore, only T stage will be used to select patients. Second, only patients with an MSS status will be included which will be determined on the biopsies, following the latest recommendations of the update of the ESMO guideline to refrain from adjuvant chemotherapy in high-risk stage II MSI colon cancer patients as the possible clinical benefit is too low [19]. This was also seen in the FOxTROT trial, where MSI status was associated with a significantly higher rate of poor/no response (96% vs. 66%, *p* < 0.0001) [20]. Using the proposed selection of patients with a MSS cT3–4NxM0 colon tumor, up to 26% of patients is estimated to be overtreated [21, 22].

Results from this study, in which we analyze both the pathological and radiological tumor response per CMS, will lead to improved patient stratification and clearer insight into which patients benefit from chemotherapy. This will allow us to identify the group of patients that receives chemotherapy appropriately and the group of patients that may not benefit from the current treatment regimen. Future studies should focus on whether chemotherapy can be withheld in this patient group or on the development of new therapies to improve patient outcome.

## Data Availability

The datasets used and/or analyzed during the current study are available from the principal investigator on reasonable request. Results will be communicated via PLCRC, presentations at international conferences and via publications in peer reviewed journals.

## Abbreviations

CAF: Cancer Associated Fibroblast
CAPOX: Capecitabin and Oxaliplatin
CMS: Consensus Molecular Subtype
ctDNA: Circulating Tumor DNA
CytoLym: Cytotoxic Lymphocytes
DCCG: Dutch Colorectal Cancer Group
ESMO: European Society of Medical Oncology
MEC: Medical Ethics Committee
MSI: Microsatellite Instable
MSS: Microsatellite Stable
OS: Overall Survival
PLCRC: Prospective Dutch ColoRectal Cancer cohort
RFS: Recurrence Free Survival
TRG: Tumor Response Grading
